# An educational video on long-acting reversible contraception as a counseling tool for postpartum adolescents

**DOI:** 10.1186/s40834-022-00195-8

**Published:** 2022-11-14

**Authors:** Somsook Santibenchakul, Kittithorn Thanativakul, Unnop Jaisamrarn

**Affiliations:** 1 Department of Obstetrics and Gynecology, Faculty of Medicine, Chulalongkorn University, Bangkok, Thailand; 2 Department of Obstetrics and Gynecology, King Chulalongkorn Memorial Hospital, Bangkok, Thailand; 3 Present address: 1873 Department of Obstetrics and Gynecology, Faculty of Medicine, King Chulalongkorn Memorial Hospital, Rama IV Road, Pathumwan, Bangkok, 10330 Thailand

**Keywords:** Teenage pregnancies, Long-acting reversible contraception (LARC), Counseling, Educational video, Postpartum adolescents

## Abstract

**Objective:**

To assess the effects of using an animated local language educational video to counsel postpartum adolescents on the benefits and use of long-acting reversible contraception (LARC).

**Materials and methods:**

A two-stage, single group, experimental study was conducted. A total of 124 female adolescents aged < 20 years who had given birth within the last six weeks participated in the study. An educational video and a questionnaire were developed and validated. Participants were asked to fill out a questionnaire on basic demographic data, contraception preferences, and 10 true/false statements to test general contraceptive knowledge, after which they were shown an educational video in a private room. Following this, participants completed the second part of the questionnaire that assessed their knowledge using the same true/false statements and contraception preferences administered earlier.

**Results:**

The mean age (standard deviation) of participants was 18.1 (1.5) years. Participants’ mean age (SD) at the time of their first sexual intercourse was 16.2 (1.6) years. Among the 124 participants, 31 (25%) indicated that they would use LARC before viewing the educational video. After viewing the educational video, this number increased to 48 (38.7%). The participants’ knowledge score was independently associated with their preference to select LARC (adjusted odds ratio 1.46, 95% confidence interval 1.09- 1.97).

**Conclusion:**

This study demonstrated that counseling tools such as animated local language educational video might effectively improve contraceptive knowledge and the preference for LARC in postpartum adolescents. An educational video regarding LARC could be used as a counseling tool for postpartum adolescents.

## Introduction

Unintended pregnancy in teenagers is a major problem worldwide. Each year, at least 10 million unintended pregnancies occur among adolescent girls aged 15–19 in developing regions [1]. Pre-term labor, low birth weight, and unsafe abortion in teenage pregnancies contribute to high rates of maternal and child morbidity and mortality [2]. The World Health Organization (WHO) reported that an estimated 3.9 million unsafe abortions occur in girls aged 15–19 years annually [1]. These pregnancies may stem from not using or using less effective contraception. Evidence shows that comprehensive contraceptive counseling can affect young women's decisions on long-acting reversible contraception (LARC) initiation, a cost-saving strategy for preventing unintended pregnancy [3–5]. The American College of Obstetricians and Gynecologists (ACOG) recommends LARC as a first-line contraceptive for adolescents [6], while another study found that effective counseling could improve contraceptive use [7]. Social media has also been used as an adjunct to traditional, in-office counseling and was shown to improve the patients’ contraceptive knowledge as well as preference for LARC [8]. However, there is limited data regarding the implementation of an educational video as a counseling tool [9]. The primary objective of this study was to assess the effectiveness of an educational video in helping sexually active postpartum adolescents select the most appropriate contraceptive method based on their preferences. The secondary objective was to assess the factors associated with the preference for LARC in sexually active postpartum adolescents. The benefits of using educational videos are that they are easier to use, consistently reproducible, have no variations as seen in traditional, in-office counseling, and can be used in almost any setting with low-cost electronic devices. We hypothesized that viewing an animated educational video would give postpartum adolescent mothers contraceptive knowledge, encouraging them to select highly effective contraception [10].

## Materials and methods

### Study design

This was a two-stage, single-group, experimental study conducted at the Family Planning and Reproductive Health Clinic of the King Chulalongkorn Memorial Hospital. During the study period, LARC, including contraceptive implant(s) and copper intrauterine devices (IUDs), became freely available through the national health care program for all adolescents. We recruited women aged < 20 years who had given birth within the last six weeks and were fluent in Thai. Women who had an absolute contraindication for one of the following contraceptives were excluded: combined/progestin oral contraceptive pills, depot medroxyprogesterone acetate (DMPA), contraceptive implant (s), and copper IUDs. Based on these criteria, a total of 124 female adolescents participated in the study.

### Ethical approval

This study was conducted in accordance with the principles of the Declaration of Helsinki. This study was approved by the Ethics and Institutional Research Committees of the Faculty of Medicine (IRB No.135/57) and registered in the Thai Clinical Trials Registry (TCTR) #: TCTR20150715001. Written informed consent was obtained in Thai from participants before starting any procedures.

### Study protocol and data collection

An animated 10-min Thai language educational video was developed according to the WHO's family planning handbook [11]. The contents of the educational video provided the following information: 1) outcomes and morbidities of teenage pregnancies, such as pre-term labor and low-birth weight; 2) types of contraception available, including tubal resection and short- and long-acting reversible contraception; 3) pros, cons, and failure rates of each contraceptive method; 4) LARC insertion procedures; and 5) the average cost per month for each contraceptive method. We provided participants with information regarding freely available LARC through the national health care program for all adolescents. The contraceptive methods covered in the educational video were as follows: combined/progestin oral contraceptive pills, DMPA, male condoms, contraceptive implant(s), and copper IUDs. The questionnaire used in this study was divided into two parts. The first part of the questionnaire was administered before the educational video was viewed in a private room. Basic demographic data and characteristics of the participants were collected, including their age, educational level, income, and marital status. The questionnaire also asked about participants’ contraceptive preferences and history of contraceptive use. An additional 10 true/false statements were used to assess participants’ general contraceptive knowledge. Answers were given a score of 1 for correct, 0 for incorrect. The possible lowest-to-highest knowledge scores ranged from 0–10. The second part of the questionnaire was administered after the educational video was viewed. The same 10 true/false statements from the first part of the questionnaire were repeated. Participants were also asked which contraceptive method they would like to use after viewing the educational video. The educational video and questionnaires were validated by two independent family planning experts (SS and UJ).

The sample size was calculated based on a pilot study of 30 postpartum adolescents conducted at the same institution. To achieve 80% power at a 5% significance level, a sample size of 124 participants was required.

### Statistical analysis

Data analysis was performed using the IBM SPSS for Windows (version 17.0; IBM Corporation, Armonk, NY). Means, standard deviations (SDs), and percentages were used to describe the demographic characteristics. McNemar’s test was used to compare the selection of LARC before and after viewing the educational video. Multivariable models were analyzed, which included variables that were potential confounders based on both previous literature and univariate analysis (p ≤ 0.2) [12, 13]. Odds ratios (ORs), adjusted odds ratios (aORs), *p*-values, and 95% confidence intervals (CIs) were calculated to estimate factors that were independently associated with preference to select LARC. Statistical significance was set at *p* < 0.05.

## Results

**Fig. 1:**
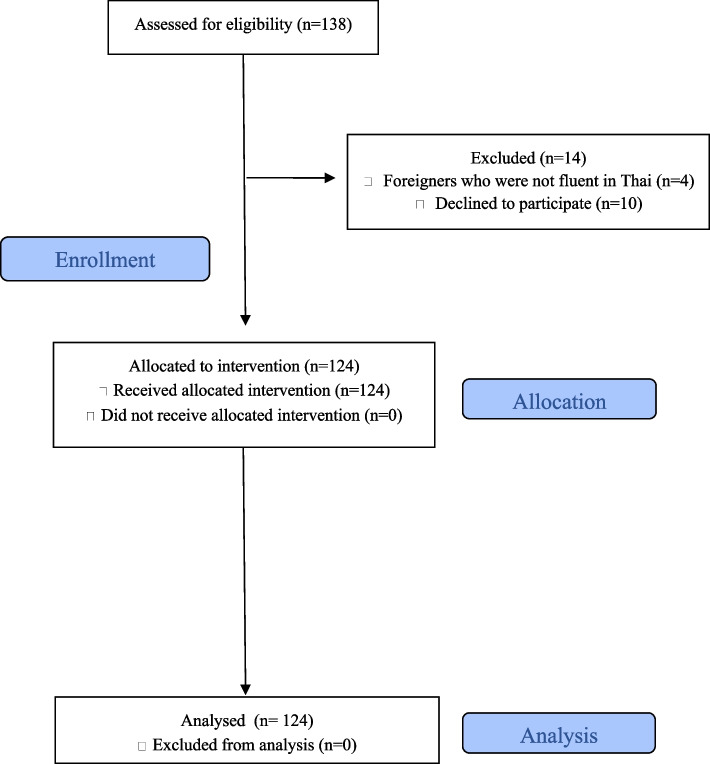
Study flow

A total of 124 female adolescents were enrolled in the study. The study flow is shown in Fig. 1. The sociodemographic and obstetric characteristics are described in Table 1. The mean age (SD) of the postpartum adolescents who participated in the study was 18.1 (1.5) years. Participants’ mean age (SD) at the time of their first sexual intercourse was 16.2 (1.6) years. Thirty-five percent of the participants had their first sexual intercourse at age ≤ 15 years. Approximately three-quarters (92/124, 74.2%) of the participants had not completed their high school education. The majority of participants (52/124, 41.9%) were students. Half of the participants’ (63/124, 50.8%) parents were either separated or dead. More than half of the participants (85/124, 68.5%) used contraception during their first sexual intercourse. Of these, the following contraceptive methods were used: 61.2% (52/85) used combined oral contraceptive pills, 28.8% (15/52) used emergency pills, and 28.8% (15/52) used male condoms. Twenty-four participants (24/124, 19.3%) had more than one child and 54% (13/24) of them were pregnant within two years of the previous birth.

**Table 1:** Socio-demographic and obstetric characteristics of the post-partum adolescent mothers at the King Chulalongkorn Memorial Hospital (*N* = 124)

Characteristics	Number (%)
Highest educational level attained	
Primary school	16 (12.9)
Lower secondary school	76 (61.3)
Upper secondary school	21 (16.6)
Vocational/ Diploma	11 (8.9)
Occupation	
Student	52 (41.9)
Employee	46 (37.1)
Housewife	21 (16.9)
Business	5 (4)
Income (US dollars per month)	
0–300	101 (81.5)
> 300	23 (18.5)
Source of income	
Family	81 (65.3)
Work	43 (34.7)
Contraception use at first sexual intercourse	
Yes	85 (68.5)
No	39 (31.5)
Number of children	
1	100 (80.6)
2	23 (18.5)
3	1 (0.8)
Pregnancy intention	
Intentional	47 (37.9)
Unintentional	77 (62.1)
Reason for unintended pregnancy	
Contraceptive failure	32 (41.6)
No contraceptive use	45 (58.4)
Health insurance	
Yes	87 (70.2)
No	37 (29.8)

Approximately 75% (93/124) of the participants initially selected non-LARC, but after viewing the educational video, 21.5% (20/93) changed their minds and reported they would prefer to use LARC.. However, three participants (3/31, 9.6%) who initially said they would prefer to use LARC before viewing the educational video later changed their preference to a non-LARC method because they were afraid of the pain associated with the procedure. The mean (SD) contraceptive knowledge scores increased from 6.8 (1.2) points to 7.9 (1.4) points after viewing the educational video. Participants who selected LARC after viewing the educational video said they had changed their opinion due to benefits such as effectiveness (35/48, 72.9%) and the need for long-term contraception (28/48, 58.3%). Half of the participants (40/76, 52.6%) who did not select LARC stated that they were afraid of pain associated with the procedure. Approximately 35% (27/76) of the participants stated that they had some concerns about the side effects of LARC.

Univariable analysis showed that knowledge score after counseling/viewing the educational video was significantly associated with the preference to use LARC (Yes) (OR 1.42, 95% CI 1.07—1.88, *p* = 0.02) as described in Table 2. When confounding variables were adjusted in the multivariable model, the knowledge score after viewing the educational video was still significantly associated with the preference to use LARC (Yes) (aOR 1.46, 95% CI 1.09—1.97, *p* = 0.01).

**Table 2:** Logistic regression analysis of factors associated with the preference to use the LARC method after viewing an educational video^a^ (*N* = 124)

**Variables**	**Unadjusted models**			**Adjusted models**^b^		
	**Odds ratio**	**95% CI**	***p*-value**	**Adjusted odds ratio**^b^	**95% CI**	***p*-value**
Age	0.80	0.64–1.03	0.07	0.86	0.66–1.13	0.28
Source of income - Family vs. Work	0.57	0.26–1.25	0.16	0.51	0.20–1.27	0.15
Health insurance support - No vs. Yes	0.57	0.25–1.30	0.18	0.45	0.19–1.12	0.09
Pregnancy intention - Intentional vs. Unintentional	1.86	0.86–4.03	0.11	1.55	0.67–3.60	0.30
Knowledge scores after viewing the educational video	1.42	1.07–1.88	0.02	1.46	1.08–1.97	0.01

^a^Dependent variable—the preference to use LARC method vs. non LARC method

^b^Adjusted for age, source of income, health insurance support, pregnancy intention

## Discussion

Our study shows that the use of an animated 10-min Thai language educational video as an adjunct to the traditional in-office counseling method can significantly increase the knowledge about LARC and contribute to the subsequent preference for LARC methods. This finding is consistent with the results of a randomized controlled clinical trial conducted by Burapasikarin et al., which showed that an educational video on LARC can increase its utilization during the postpartum period [9], Another study showed that reading the information on LARC out loud to the participants and giving them a written document on LARC did not increase their preference for LARC [14]. Likewise, the use of an educational script on the intrauterine device and contraceptive implant did not increase the preference for LARC postpartum [14]. This indicates that providing information on LARC without using other educational techniques may not affect the preference for LARC. Additional techniques, such as educational videos, can significantly affect the preference for LARC as it can help the viewers concentrate more on the knowledge content and be more engaged in the topic.

The results of the multivariable analysis showed that participants’ general contraceptive knowledge regarding LARC might have affected their preference for LARC. Providing more information on LARC in layman’s terms may help increase the preference for selecting this contraceptive method. This finding corroborates previous reports that improved knowledge of LARC is associated with its increased use [15–17].

Our analysis shows that sources of income and health insurance were not associated with LARC selection. One possible explanation is that LARC is free of charge in our setting. This result may not be generalizable as the cost can be a barrier in other locations and could decrease the preference for LARC [18]. The results of our study are most applicable to postpartum adolescents in comparable settings in Southeast Asian countries. Generalizable to other groups of different ages, educational attainment, and cultural contexts may be limited. These factors might affect clients' health literacy, contributing to their decision to initiate contraception. Another limitation is that our analysis was restricted to participants’ preferences for using LARC. However, information regarding LARC initiation was not available, as most participants preferred to delay contraceptive initiation until the postpartum follow-up visit.

## Conclusion

This study successfully demonstrated the effectiveness of using an animated 10-min Thai language educational video as a counseling tool to significantly increase the preference for LARC among postpartum mothers. Additional studies with longer follow-up periods are necessary to evaluate how this educational video subsequently affects patient decision-making to choose LARC over other methods of contraception.

## Data Availability

The data and material that support the findings of this study are available upon request.
